# In vitro and in vivo comparative study of a novel ^68^Ga-labeled PSMA-targeted inhibitor and ^68^Ga-PSMA-11

**DOI:** 10.1038/s41598-021-98555-y

**Published:** 2021-09-27

**Authors:** Huanyu Chen, Ping Cai, Yue Feng, Zhanliang Sun, Yinwen Wang, Yue Chen, Wei Zhang, Nan Liu, Zhijun Zhou

**Affiliations:** 1 The Department of Nuclear Medicine, Affiliated Hospital of Southwest Medical University, Jiangyang District, Luzhou, Sichuan China; 2 Nuclear Medicine and Molecular Imaging Key Laboratory of Sichuan Province, Jiangyang District, Luzhou, Sichuan China; 3 Academician (Expert) Workstation of Sichuan Province, Jiangyang District, Luzhou, Sichuan China; 4 Department of Pharmaceutics, School of Pharmacy, Southwest Medical University, Jiangyang District, Luzhou, Sichuan China

**Keywords:** Biomarkers, Molecular medicine, Oncology, Urology

## Abstract

^68^Ga-radiolabeled small molecules that specifically target prostate-specific membrane antigen (PSMA) have been extensively investigated, and some of these tracers have been used in the diagnosis of prostate cancer via ^68^Ga-positron emission tomography (^68^Ga-PET). Nevertheless, current ^68^Ga-labeled radiotracers show only fair detection rates for metastatic prostate cancer lesions, especially those with lower levels of prostate specific antigen (PSA), which often occurs in the biochemical recurrence of prostate cancer. The goal of this study was to design and synthesize a new PSMA-targeted radiotracer, ^68^Ga-SC691, with high affinity for prostate cancer cells and excellent pharmacokinetics. To this end, structural optimization was carried out on the bifunctional group, target motif, and linker while the high affinity targeting scaffold remained. To explore its potential in the clinic, a comparative study was further performed in vitro and in vivo between ^68^Ga-SC691 and ^68^Ga-PSMA-11, a clinically approved tracer for PSMA-positive prostate cancer. SC691 was radiolabeled to provide ^68^Ga-SC691 in 99% radiolabeling yield under mild conditions. High uptake and a high internalization ratio into LNCaP cells were observed in in vitro studies. In vivo studies showed that ^68^Ga-SC691 had favorable biodistribution properties and could specifically accumulate on PSMA-positive LNCaP xenografts visualized by micro-PET/CT. This radiotracer showed excellent PET imaging quality and comparable, if not higher, uptake in LNCaP xenografts than ^68^Ga-PSMA-11.

## Introduction

Prostate cancer (PCa) is the most common form of cancer in males in the USA. It is estimated that there will be more than 190,000 new cases of prostate cancer and an estimated 33,000 deaths from this disease in the USA in 2020 alone, according to the National Cancer Institute^1^. Statistically, even though prostate cancer cells in most PCa cases remain local, approximately 10% of the total number of PCa patients will suffer from metastasis or biochemical recurrence^2^. Advanced techniques such as computed tomography (CT) and magnetic resonance imaging (MRI) provide high-contrast and high-resolution cancer images; however, imaging outcomes from these methods often suffer from either a lack of functional information or the explicit explanation needed from the corresponding professionals^3–5^. Additionally, it is difficult to detect microscale lesions, which are often observed in postoperative examinations. Positron emission tomography (PET), as a functional imaging method, together with targeting radiotracers, has been widely used in the clinic due to its precision and ultrahigh sensitivity^6^. To date, PET/CT and PET/MRI fusion imaging as novel multimodality technology, which combines the benefits of anatomical information from CT imaging or MRI and functional information from PET, have become one of the most powerful imaging methods in disease diagnosis and single cell tracking^7, 8^.

PCa possesses unique pathophysiological and biological characteristics. A large number of studies have found that the overexpression of transmembrane proteins, namely, prostate-specific membrane antigen (PSMA), also known as glutamate carboxypeptidase, dominates in over 90% of prostate carcinomas and the tumor-associated endothelium of various solid cancer tissues^9, 10^. Along with other properties, such as over 94% of the amino acids of the transmembrane protein being located outside of the membrane^11^, PSMA has become one of the most extensively studied drug targets and shows great promise in prostate cancer cell detection^12–14^.

**Figure 1:**
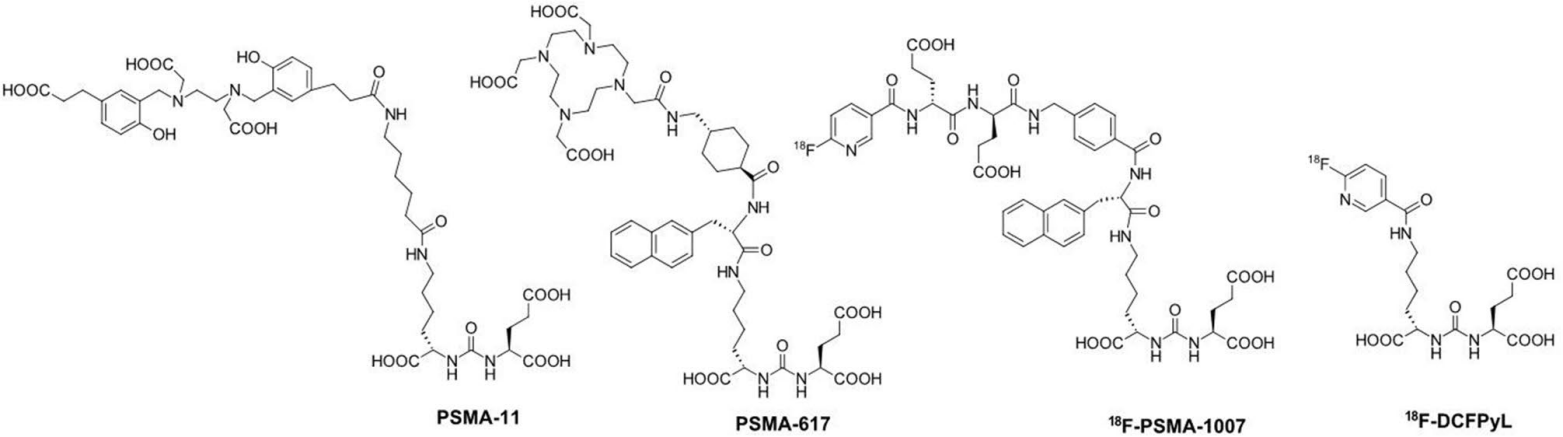
The most often-used precursors based on the Lys-urea-Glu scaffold for PCa detection.

Even though macromolecules such as antibodies were introduced a few decades ago^15^, due to the outstanding merits of small molecules, such as their existence as a pure substance, definitive structure, and clear structure–activity relationships, small molecule PSMA inhibitors have mostly been investigated^16^. According to targeting scaffolds, small molecule PSMA inhibitors fall into one of three major families: (1) thiols^17^; (2) phosphonate-, phosphate-^18^, and phosphoramidates; and (3) ureas^19^. Among these inhibitors, small molecules with a Lys-urea-Glu scaffold as a pharmacophore were found to be able to accumulate in prostate cancer cells specifically and efficiently. Thus, a series of Lys-urea-Glu-based PSMA-targeted imaging agents with favorable biological properties emerged to visualize PCa lesions, such as ^18^F-DCFPyL, ^18^F-PSMA-1007, ^68^Ga-PSMA-11^20^, and ^68^Ga-PSMA-617^21–24^ (Fig. 1). Until now, no PET agents have been approved for clinical use in PCa diagnosis except for ^68^Ga-PSMA-11, which was approved in December 2020^25^. Unlike ^18^F-radiolabeled tracers, which need cyclotrons to produce ^18^F and complex procedures and facilities for the synthesis of ^18^F-radiopharmaceuticals, the ready availability and efficient complexing characteristics of ^68^Ga(III), together with its favorable biological properties, have made the use of ^68^Ga-based PSMA-targeted PET procedures grow rapidly, as evidenced by an increasing number of clinical trials^26–30^. Among them, ^68^Ga-PSMA-11 is the most investigated and the only PCa radiotracer approved by the FDA. Currently, it is routinely used in the diagnosis, treatment evaluation, and staging of PCa.

Herein, we present the synthesis and in vitro and in vivo study of a novel and PSMA-targeted radiotracer, ^68^Ga-SC691. We further evaluated its affinity and specificity to PSMA-positive tumors by comparing its pharmacokinetics and PET imaging abilities with those of ^68^Ga-PSMA-11. ^68^Ga-SC691 displayed favorable pharmacokinetics and excellent uptake in PSMA-positive tumors, indicating that ^68^Ga-SC691 may serve as a new PET tracer for prostate cancer.

## Results

### Chemical, radiochemical synthesis and characterization

**Scheme 1.**
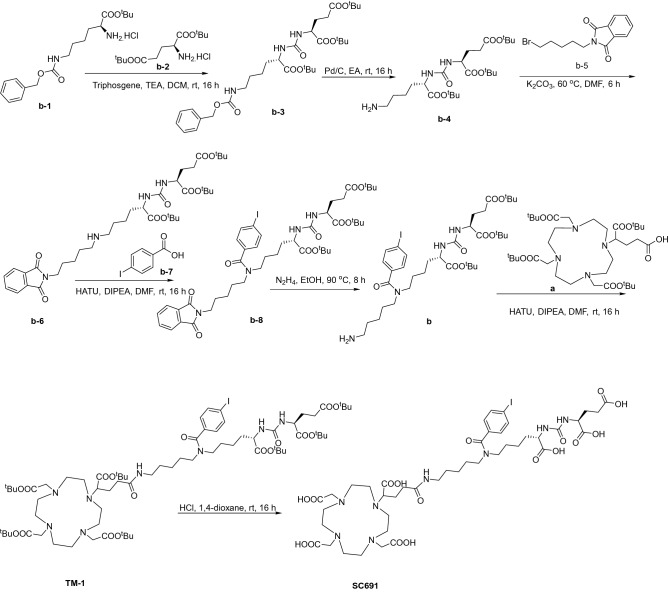
Synthesis of SC691.

We first constructed the urea-based scaffold **b-3** bearing protected glutamine and lysine residues followed by hydrogenation to provide **b-4** with a combined yield of 51.1%. Converting **b-4** into **b-6** requires attention to the ratio of reactants; otherwise, a disubstituted compound will be the major product instead of the monosubstituted compound **b-6**. Compound **b-6** was produced in a yield of 77.7%. With **b-6** in hand, we further converted the secondary amine into tertiary amine **b-8** with a *para*-iodo benzoyl moiety. After reduction of **b-8**, conjugation between **a** and **b** was performed to provide **TM-1**. The target molecule SC691 was obtained via deprotection of TM-1 in dilute acid in a yield of 39.1% (Scheme 1, Figure S1, Figure S2). The multistep reactions were conducted with a total yield of 2.8%.

**Figure 2:**
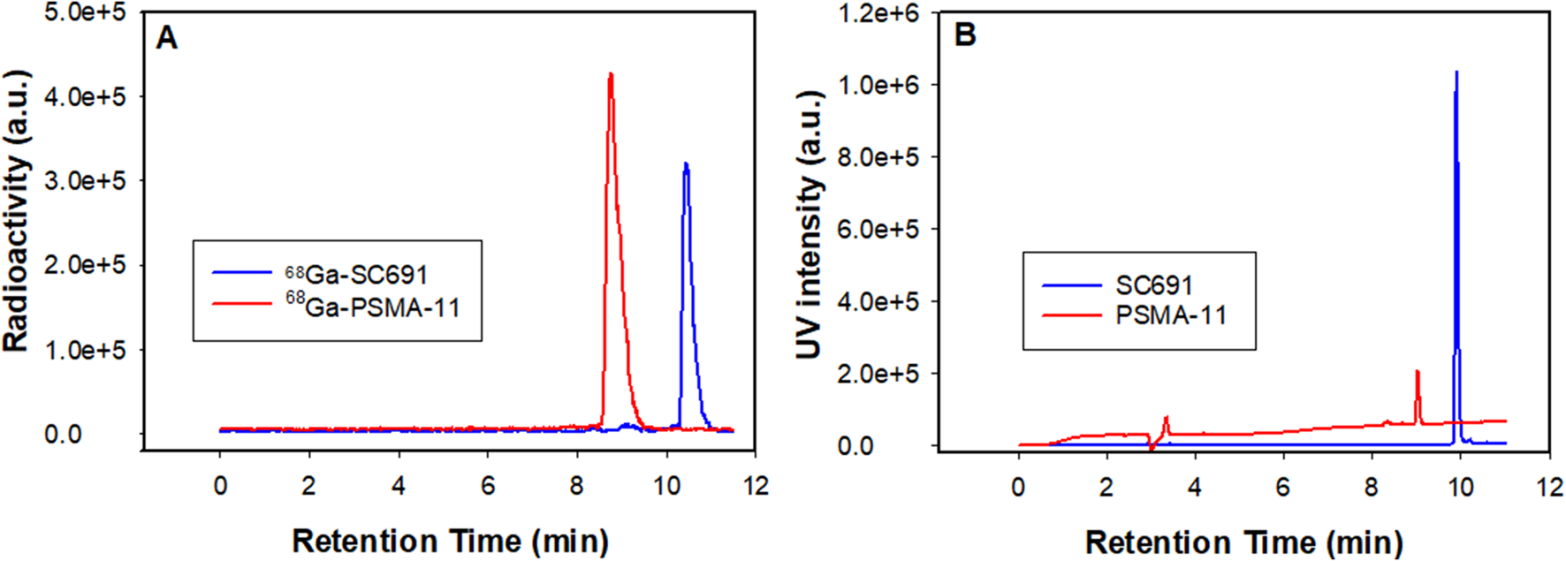
HPLC chromatograms of ^68^Ga-PSMA-11 (red line) and ^68^Ga-SC691 (blue line) (**A**) and PSMA-11 (red line) and SC691 (blue line) (**B**).

Radiolabeling of SC691 with ^68^Ga(III) eluate is straightforward. It may be performed in NaAc/HAc buffer or HEPES buffer (pH 4.0–5.0) at ambient temperature or a temperature as high as 95 °C in high radiolabeling yield (~ 95–99%) and radiochemical purity (> 98%) after purification, as analyzed using analytical reversed-phase high performance liquid chromatography (RP-HPLC, equipped with a γ detector) with a specific activity as high as 18.8 MBq/μg (Fig. 2). The retention time increased from 9.9 to 10.4 min after SC691 was radiolabeled with ^68^Ga(III). In the cases of PSMA-11 and ^68^Ga-PSMA-11, the retention times were 8.7 min and 9.0 min, respectively. Thus, SC691 displayed a longer retention time on HPLC, and an increase in the retention time was observed for both precursors upon radiolabeling.

### Lipophilicity and stability

**Figure 3:**
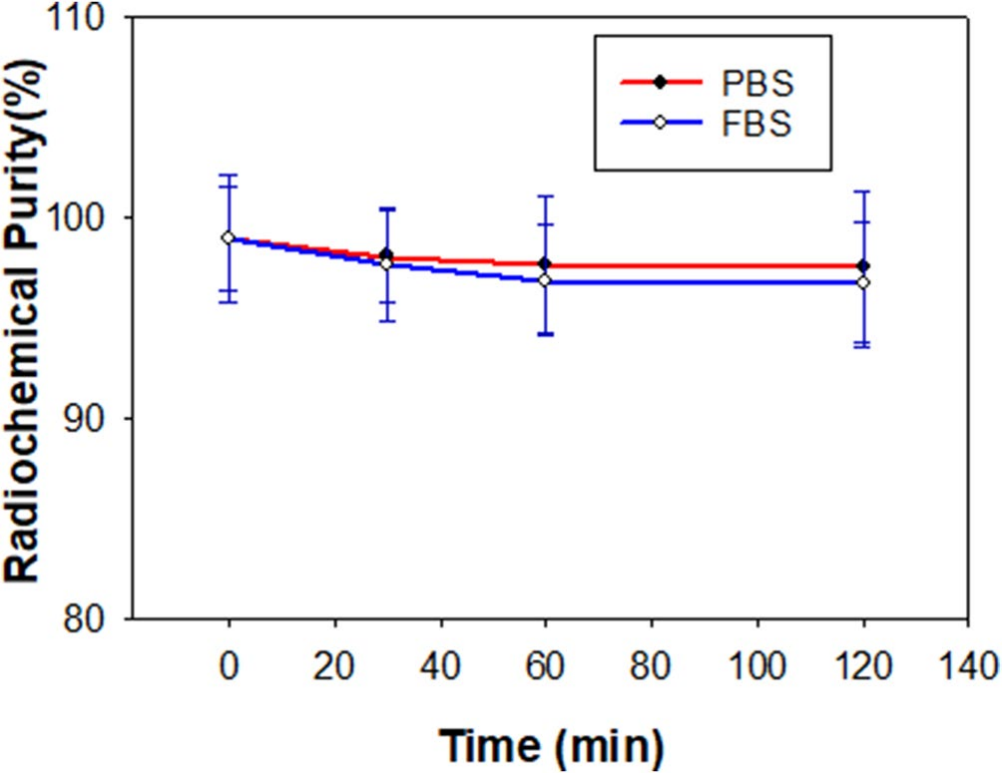
Stability of ^68^Ga-SC691. Radiochemical purity was determined in PBS and FBS at 0 min (initial point), 30 min, 60 min, and 120 min.

We further investigated the hydrophilicity and stability of ^68^Ga-SC691. The partition coefficient (Log P) between octane and phosphate-buffered saline (PBS) is usually used to measure the hydrophilicity of a compound. ^68^Ga-SC691 and ^68^Ga-PSMA-11 had Log P values of − 3.530 ± 0.086 and − 2.91 ± 0.06^31^, respectively (Table 1). These data indicated that ^68^Ga-SC691 is more hydrophilic than ^68^Ga-PSMA-11. A stability study of ^68^Ga-SC691 was performed both in PBS and fetal bovine serum (FBS) at 37 °C in triplicate. The radiotracer showed time-dependent stability in both systems, but after 2 h, its excellent stability was revealed, as over 96% of the ^68^Ga-SC691 remained unchanged as determined by its retention time on analytical radio-HPLC (Fig. 3).

**Table 1:** Analytical data of SC691, ^nat^Ga-SC691, and PSMA-11.

Compound code	Chemical formula	MW (g/mol)	m/z^1^	tr (min)	Radiochemical purity (%)	Log D
SC691	C_43_H_68_IN_8_O_17_	1092.92	1093.3472	9.91	N.A.	N.A.
^nat^Ga-SC691	GaC_43_H_65_IN_8_O_17_	1159.05	N.A.	10.11	99%^2^	− 3.530 ± 0.086^3^
PSMA-11	C_44_H_62_N_6_O_17_	946.99	947.4257	9.04	96%^5^	− 2.91 ± 0.06^4^

^1^Mass spectrometry data detected as [M+H]^+^

^2^Value for radiochemical purity measured by RP-HPLC. An Agilent analytical column (250 × 4.6 mm) was utilized with mobile phases consisting of 0.1% TFA in water (**A**) and ACN (**B**). For analytical runs, a linear gradient of solvent A (90–10% in 15 min) in solvent B at a flow rate of 1.0 mL/min was used for a 15 min run.

^3^Obtained from ^68^Ga-SC691. See the detailed method description in the “Materials and methods” section.

^4^This value is for ^68^Ga-PSMA-11 from the literature^31^.

^5^The purity of PSMA-11 was obtained from the ABX GmBH certificate of this compound.

Another important aspect of a radiopharmaceutical is its radiochemical stability, which determines whether it is worth carrying out further in vivo and in vitro studies on a radioligand. ^68^Ga-SC691 showed excellent stability both in PBS and in FBS after incubation for 2 h at 37 °C. By the end of the 2 h incubation in PBS and FBS, ^68^Ga-SC691 maintained radiochemical purities of 97.9% and 97.5%, respectively.

### Cell affinity studies

**Figure 4:**
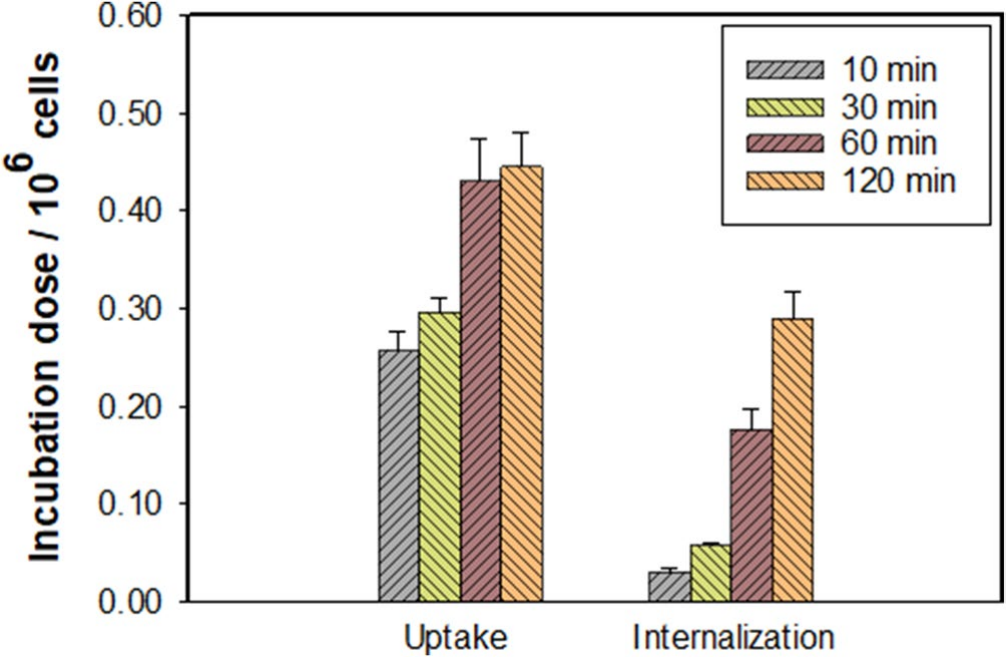
The uptake and internalization of ^68^Ga-SC691 in LNCaP cells (~ 200,000 cells/well, normalized to 10^6^ cells) at 10 min, 30 min, 60 min, and 120 min.

Uptake and internalization experiments of ^68^Ga-SC691 in LNCaP cells revealed high uptake and internalization rates (Fig. 4). Both internalization and uptake of ^68^Ga-SC691 displayed a time-dependent pattern and maintained an increasing trend for 120 min. Interestingly, uptake of ^68^Ga-SC691 nearly reached saturation at 60 min, and only a slight increase was observed after this time. On the other hand, internalization experienced a relatively fast increase throughout the whole experimental period. A blocking experiment with 2-PMPA was conducted to demonstrate the specificity of ^68^Ga-SC691 toward PSMA in vitro. The cellular uptake of ^68^Ga-SC691 was nearly completely blocked by 2-PMPA (data not shown).

### Biodistribution

**Figure 5:**
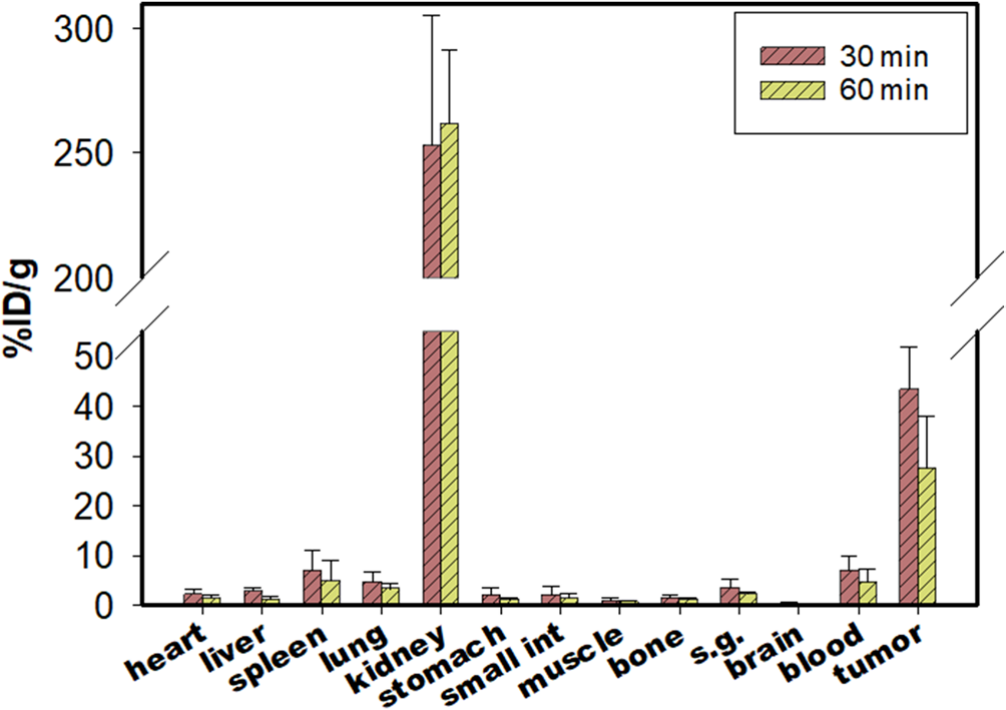
Organ biodistribution of ^68^Ga-SC691 expressed as % ID/g tissue at 30 min and 60 min postinjection. Data are expressed as the means ± SD (n = 5). *s.g.* salivary glands, *small int* small intestine.

The results of the biodistribution study of ^68^Ga-SC691 in LNCaP mice bearing prostate cancer (NOD/SCID) were decay-corrected and reported as the percentage of the injected activity per gram of tissue mass (% ID/g) and presented as the average ± standard deviation (SD). As summarized in Fig. 5, organ distribution with ^68^Ga-SC691 revealed high specific uptake in LNCaP tumors with values of 43.41 ± 8.39% ID/g at 30 min and 27.59 ± 10.38% ID/g at 60 min. This high accumulation of radioactivity was maintained over 2 h of measurements (observed from micro-PET/CT). A similar trend in uptake was observed for the kidneys.

The uptake of ^68^Ga-SC691 was shown to be specific by coinjection of ^68^Ga-SC691 together with 2-PMPA, demonstrating reduced tumor uptake by 30.55% ID/g at 60 min postinjection (12.86 ± 2.80% ID/g, n = 2). Biodistribution studies of ^68^Ga-SC691 also demonstrated pronounced uptake in the kidneys and slight uptake by the liver, probably suggesting both hepatobiliary and kidney excretion pathways. However, the much higher uptake of ^68^Ga-SC691 shown by the kidneys than the liver suggests that the kidneys contributed most significantly and are thus the principal excretion organs. Tumor-to-background ratios were determined to be 5.86 (tumor/blood) and 34.49 (tumor/muscle) at 1 h postinjection. Consistent with other PSMA-specific radioligands, no significantly reduced uptake, except for that in the kidneys, occurred in normal organs, as seen in blocking experiments.

### Micro-PET/CT imaging

**Figure 6:**
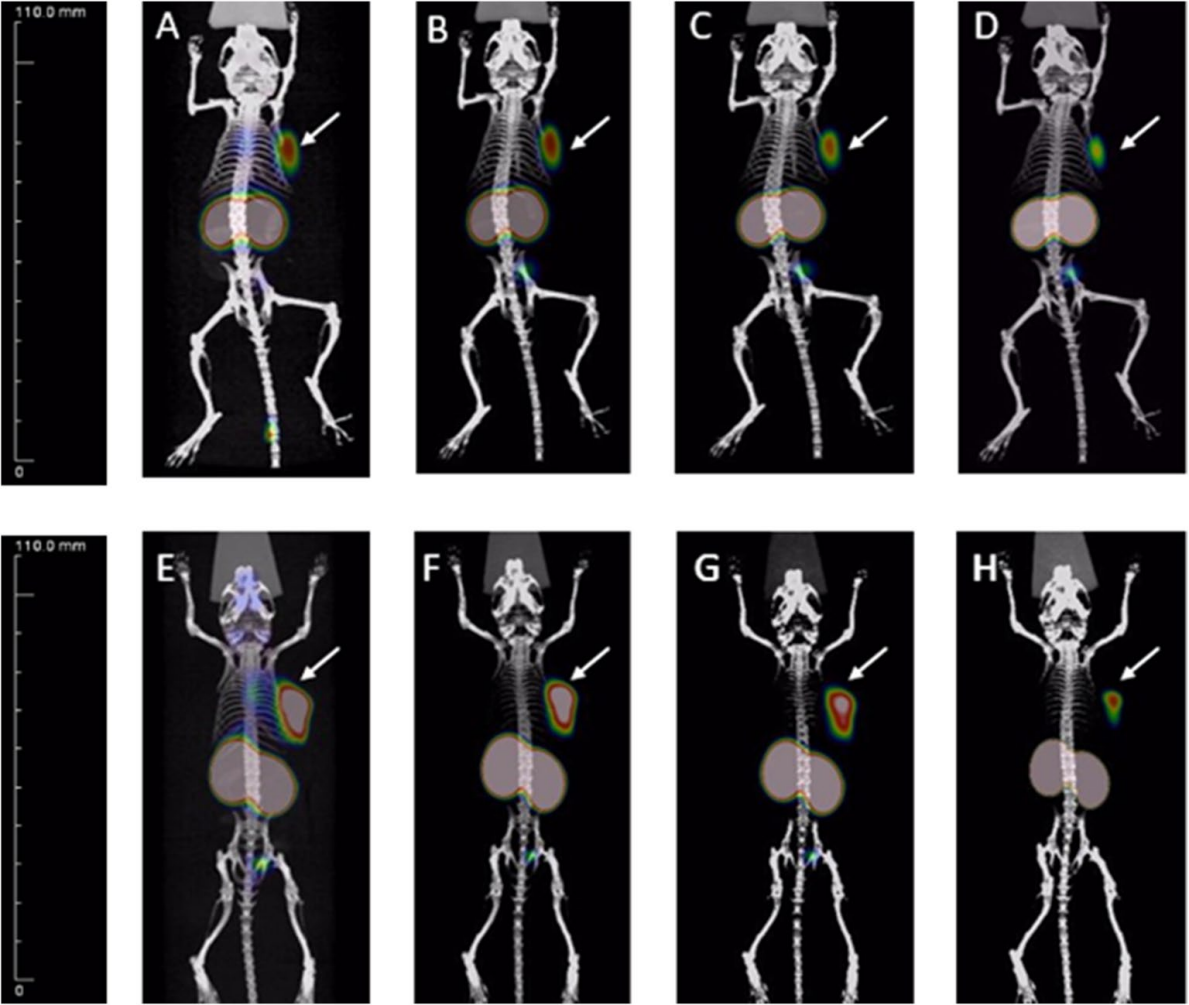
Maximum intensity projection of the whole-body coronal micro-PET/CT images of a NOD/SCID male mouse bearing a LNCaP tumor xenograft (white arrow). The tumor targeting efficacy of ^68^Ga-SC691 and ^68^Ga-PSMA-11 was demonstrated by time-dependent static scans at 5 min, 30 min, 60 min, and 120 min postinjection of ^68^Ga-SC691 (**A**–**D**) and ^68^Ga-PSMA-11 (**E**–**H**). Approximately 1.9 MBq/mouse was injected.

**Figure 7:**
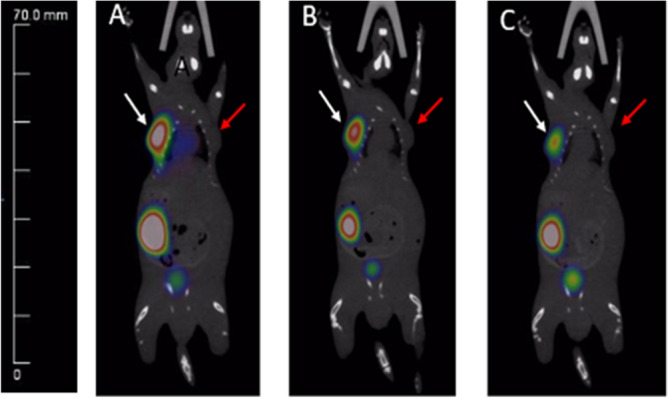
Whole-body coronal micro-PET/CT images of a NOD/SCID male mouse bearing a LNCaP tumor xenograft in PC-3 and LNCaP inoculated xenografts (LNCaP on the left with a white arrow, PC-3 on the right with a red arrow) postinjection of ^68^Ga-SC691: 30 min (**A**), 60 min (**B**), 120 min (**C**).

**Figure 8:**
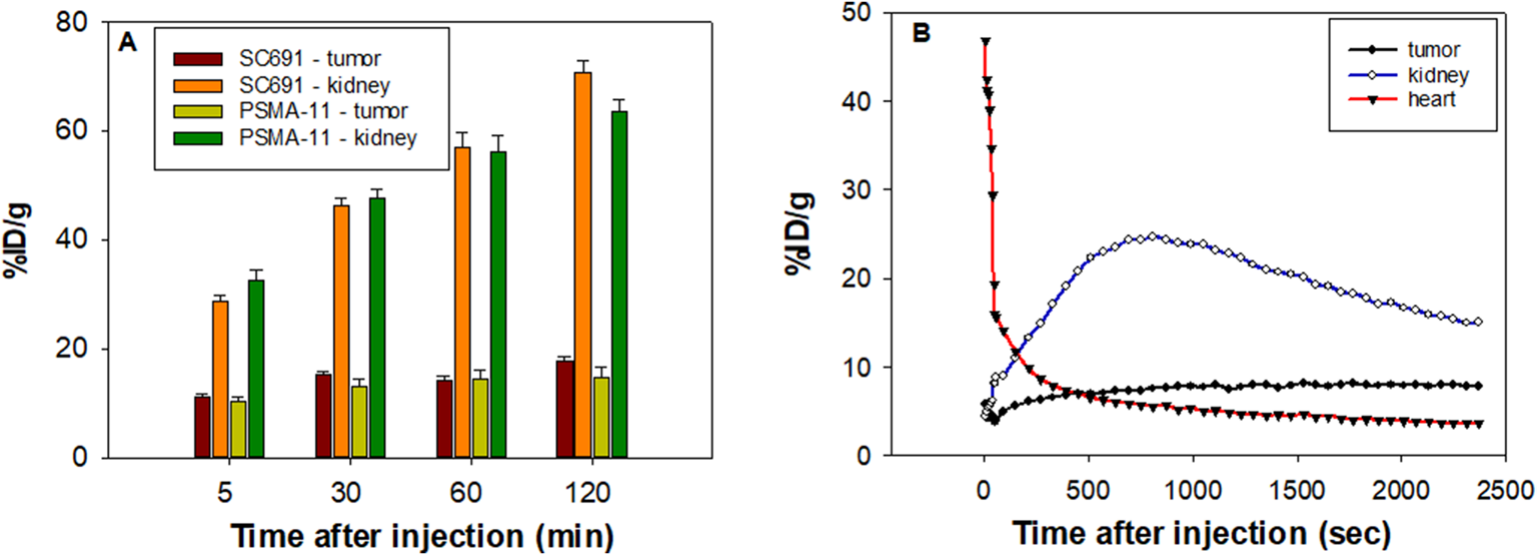
The % ID/g obtained from the whole-body coronal micro-PET/CT scans of NOD/SCID male mice bearing LNCaP tumor xenografts (**A**). The tumor-targeting efficacy of ^68^Ga-SC691 was demonstrated by dynamic micro-PET scanning (**B**).

Next, whole-body micro-PET/CT imaging was carried out for ^68^Ga-SC691 and ^68^Ga-PSMA-11 (as a reference) in intact male NOD/SCID mice (Fig. 6) bearing LNCaP tumors only or bearing both LNCaP and PC-3 tumors in opposite upper flanks (Fig. 7, Figure S4). A static imaging method was utilized for both compounds at the time points of 5 min, 30 min, 60 min, and 120 min. Following the static PET scan, a dynamic PET scan was conducted to understand the time-dependent pharmacokinetics of ^68^Ga-SC691 (Fig. 8). As a result of both the static and dynamic scans, these radiotracers were able to visualize PSMA-positive LNCaP tumors as early as 5 min postinjection but not PC-3 tumors. The micro-PET/CT images and dynamic uptake curves clearly showed the fast-targeting properties and high retention rate of ^68^Ga-SC691 at the tumor site during the 2 h experiment, which was consistent with the biodistribution results. In addition to tumors, uptake by other organs, such as the heart and kidneys, became evident during the initial 5 min, and the uptake by the heart was diminished at 30 min and thereafter. Comparative PET/CT imaging also clearly showed that ^68^Ga-SC691 had a renal clearance similar to that of ^68^Ga-PSMA-11. Quantitative data from micro-PET/CT showed that the mean % ID/g at the tumor for both ^68^Ga-SC691 and ^68^Ga-PSMA-11 continued to increase up to 2 h postinjection, and the rapid elimination of radioactivity occurred from the other organs, muscles, and blood (with a quite clear background). However, the uptake curve became flattened over time, as summarized in Fig. 8A. In comparison to ^68^Ga-PSMA-11, ^68^Ga-SC691 displayed similar uptake in both the tumors and kidneys.

## Discussion

PSMA is strongly overexpressed on prostate cancer cells and therefore has been selected as one of the most important drug targets for prostate cancer diagnosis, staging, and follow-up. PSMA inhibitors, after radiolabeling with ^68^Ga, present favorable properties, such as availability and facile complexation chemistry. Here, we report the successful synthesis of ^68^Ga-SC691 via multistep chemical reactions and complexation with the ^68^Ga (III) method. To date, Lys-urea-Glu has become the most common and important targeting motif in PSMA inhibitor structures due to its high affinity and specificity for PSMA. To manage the pharmaceutical and metabolic properties of the desired tracers, amide or secondary amine linkers produced by Lys are the most popular strategy while keeping Lys-urea-Glu untouched. In 2019, Banerjee and coworkers first reported the tertiary amination modification strategy of the amino group on the lysine side chain modified by a *p*-halo benzyl moiety and obtained several PSMA inhibitors with high binding affinity and excellent in vivo properties, which set a precedent for the tertiary amination modification strategy^32, 33^. Inspired by the quite low *Ki* value of the compound *iodo*-DCFPyL (Fig. 1) (*Ki* = 0.01 nmol), we decided to introduce a *p*-iodo benzoyl moiety onto the lysine residue of the Lys-urea-Glu scaffold to verify the possibility of obtaining a PSMA inhibitor with a high affinity through the tertiary amination strategy. On the other hand, the macrocyclic chelating agent with four acetate donor arms demonstrated higher tumor uptake and retention in vitro and in vivo; thus, DOTAGA, but not DOTA, was selected as the chelating agent due to its favorable properties. A shorter or longer linker with > 4 methylene groups was reported to show no significant impact on tumor uptake and retention. Together with the chelating agent and *p*-iodo benzoyl moiety, SC691 was found to have excellent in vitro and in vivo properties.

The synthesis of SC691 is quite straightforward except for the conversion of the primary amine into a secondary amine, in which a dimer was formed. To enhance the yield of the monosubstituted product, the molar ratio and temperature should be carefully controlled. Compared with DOTA, DOTAGA has one more carboxylic acid group, providing an extra complexation site for Ga(III). The complexation chemistry of DOTAGA is easier than that of DOTA, so this reaction can be carried out at lower temperatures while achieving high radiochemical yields. This tracer was quite stable in PBS and FBS with over 96% intact compound remaining in both media after 2 h of incubation at 37 °C.

To improve precision with regard to comparison, further purification by HPLC was applied to remove the unlabeled precursor (SC691 or PSMA-11). The specificity of ^68^Ga-SC691 was confirmed by its high uptake in PSMA-positive LNCaP cells and by blocking experiments in a LNCaP-inoculated NOD/SCID mouse model (Figure S3). Accumulated radioactivity was not observed on PC-3 tumors but it was observed on LNCaP tumors. Upon coinjection of the PSMA inhibitor 2-PMPA, uptake of ^68^Ga-SC691 was completely blocked, so quite low radioactivity was detected, which indicated that the novel tracer had exceptional specificity for PSMA-positive tumors. Biodistribution also showed that the tumor was the major site and that the kidneys played a critical role as a more important excretion organ than the liver. Tumor retention of ^68^Ga-SC691 was maintained at a high level for over 2 h postinjection, which is consistent with the in vitro cell uptake and internalization results. Interestingly, the chelator DOTAGA exerted an evident impact on the properties of ^68^Ga-SC691 by changing its lipophilicity or charge. In particular, the pharmacological properties were strongly influenced by the more hydrophilic ^68^Ga-SC691.

The results obtained from the micro-PET/CT imaging experiments indicated that ^68^Ga-SC691 had a slightly higher absolute uptake than ^68^Ga-PSMA-11 in PSMA-positive tumors (Fig. 8A). An unexpected and longer blood clearance was observed for ^68^Ga-SC691, considering that ^68^Ga-SC691 is much more hydrophilic than ^68^Ga-PSMA-11 (Table 1). We proposed that structural modification not only changed the hydrophilicity but also changed the binding affinity for plasma proteins, such as albumin. To confirm this hypothesis, a serum albumin binding experiment was carried out, and it was found that introducing a *p*-iodo benzoyl moiety clearly enhanced the affinity of the compound for serum albumin, thus extending its circulation time in the blood (data not shown). This property of longer blood circulation is favored for therapeutic agents if no safety issues exist. With regard to diagnostic agents, higher accumulation at the targeted organs could also be beneficial in practice due to extended blood circulation, as the diagnostic dose of radioactivity generally does not yield significant adverse reactions but potentially improves precision prognosis.

## Materials and methods

### Precursor synthesis

The synthetic route and chemical structures of SC691 and ^68^Ga-SC691 are shown in Scheme 1. The synthesis of SC691 is quite straightforward through multistep reactions, and SC691 was purified with preparative high-performance liquid chromatography (prep-HPLC). The detailed synthesis is attached in the supporting information.

### ^68^Ga radiolabeling

Typically, 5–20 μL of SC691 (1 mg/mL in pure water) was added to a volume of 100 μL of sodium acetate/acetic acid (NaAc/HAc) buffer (NaAc/HAc = 0.5 M/0.5 M, pH = 4.45), followed by the addition of 400 μL of the ^68^Ga(III) eluate (18.5–40 MBq). The reaction mixture was incubated at 80 °C for 10 min and the radiochemical purity was determined by reversed-phase HPLC (RP-HPLC).

### ^nat^Ga complexation

A 20-fold molar excess of ultrapure ^nat^Ga(III)-chloride (Aladdin, China) in NaAc/HAc buffer (pH = 4.45, NaAc/HAc = 0.5 M/0.5 M, both ultrapure grade) (400 μL) and HCl (0.05 M, 1600 μL) was reacted with compound SC691 (1 mg/mL) for 8 h at 80 °C. The reaction mixture was purified via preparative RP-HPLC, and the product was obtained as a white solid after lyophilization.

### Radiochemical stability

The stability studies were carried out by incubating ^68^Ga-SC691 at 37 °C for 30 min, 60 min, and 120 min in either PBS buffer or FBS in triplicate. At each time point, the radiochemical purity was directly analyzed using RP-HPLC for samples in PBS buffer. Samples in FBS were pretreated before analysis. Briefly, the FBS samples were precipitated with acetonitrile, followed by centrifugation for 5 min at 10,000 rpm. Aliquots of the supernatants of the FBS samples were used for radiochemical stability analysis using RP-HPLC.

### Cell binding studies

Cell binding studies were performed using a PSMA-positive LNCaP cell line in triplicate. LNCaP cells were cultured in Gibco RPMI 1640 medium supplemented with 10% FBS and 1% streptomycin/penicillin (Thermo Fisher Scientific, USA). Cells were grown at 37 °C for 24 h in an incubator with humidified air and equilibrated with 5% CO_2_. One day prior to the experiment, after removal of the medium, the cells were washed off with trypsin-ethylenediaminetetraacetic acid (EDTA; 0.25% trypsin, 0.02% EDTA) and PBS. Cells were resuspended in the above medium, dispersed in 24-well plates and incubated under the above conditions for 24 h. The cells were washed with PBS followed by the addition of 450 μL of Gibco RPMI 1640 and 50 µL of the corresponding radioligand to each well. The plates were then incubated for 10 min, 30 min, 60 min or 120 min. To determine the uptake of the added radioactivity, the cells were washed three times with ice-cold PBS and lysed with 100 µL of 1 M NaOH. The internalized fraction was determined in the cells, which were washed with ice-cold PBS and then incubated for 10 min with acidic stripping buffer (0.05 M glycine stripping buffer in 100 mM NaCl, pH 2.8) followed by an additional washing step with ice-cold PBS. All cell samples were measured with a γ-counter (CAPRAC-t, Edmonton, Canada).

### Log D measurements

^68^Ga-SC691 was aliquoted into vials containing 500 µL of octanol and 490 µL of 0.01 M PBS (pH = 7.4). Each vial was vortexed for 5 min and centrifuged for 10 min (5000 rpm). Relative concentrations were determined by RP-HPLC (equipped with γ counter) for the octanol and PBS samples (1 mL for each sample). The Log D was determined using the following equation: Log D = Log [(γ counts in octanol phase)/(γ counts in PBS phase)]. The experiment was carried out in triplicate.

### Biodistribution and imaging studies

#### Tumor model

The human prostate cancer cell lines LNCaP and PC-3 (PSMA-negative control) were obtained from the American Type Culture Collection (ATCC). Cells were counted, and 5 × 10^6^ LNCaP or PC-3 cells in 100 μL of RPMI-1640 medium were injected subcutaneously into 4- to 5-week-old male nonobese diabetic/severe combined immunodeficiency (NOD/SCID) mice. Animals were grown under standard conditions for 3–4 weeks to allow the tumors to become established before biodistribution and imaging.

#### Biodistribution

Biodistribution studies were performed 3 to 4 weeks after LNCaP tumor cell inoculation when the tumor xenografts reached an average mass of approximately 20 ± 5 g, corresponding to a tumor diameter of 5–8 mm. SC691 was radiolabeled with ^68^Ga at a specific activity of ~ 24.5 MBq/nmol and diluted in sterilized saline (0.9% NaCl in DI water) to a final specific activity of approximately 2.8 MBq/nmol. Tumor-bearing mice were intravenously injected with 100 µL of ^68^Ga-SC691 (~ 2.8 MBq). The mice were sacrificed at 30 min, 60 min, and 120 min postinjection, the major organs were collected and weighed, and the radioactivity was measured with a γ-counter (CAPRAC-t, Edmonton, Canada). Mice were grouped at specific time points, and 5 mice were included in each group.

#### Imaging studies

The ^68^Ga-radiolabeled compound ^68^Ga-SC691 was injected into the tail veins of male mice bearing LNCaP and PC-3 tumors (~ 3.7 MBq; 100 μL). All animals were placed in the prone position for micro-PET imaging at 5 min, 30 min, 60 min and 120 min postinjection. During the imaging process, mice were anaesthetized and maintained under 2% isoflurane in oxygen at a flow rate of 2 L/min. Data analysis was performed using Inveon Research Workplace software. The experiment was repeated at least three times.

### Institutional Review Board statement

This study was approved by the Ethics Committee for Animals of Southwest Medical University (2019-06-27). All methods were carried out in accordance with relevant guidelines and regulations, and all methods were reported in accordance with ARRIVE guidelines.

## Conclusions

In summary, we report a novel ^68^Ga-labeled ligand for the noninvasive detection of prostate cancer. This ligand showed promising binding and internalization properties in vitro as well as high specific uptake and a favorable retention time in vivo. This radiotracer showed excellent PET imaging quality and uptake that was comparable with ^68^Ga-PSMA-11 in LNCaP xenografts, if not higher. The feasibility of prostate cancer imaging with ^68^Ga-SC691 is under evaluation in humans.

## Supplementary Information

Supplementary Information.
