# Supplementary material for: In vitro and in vivo comparative study of a novel 68Ga-labeled PSMA-targeted inhibitor and 68Ga-PSMA-11
Source: Sci Rep. 2021 Sep 27;11:19122. doi: 10.1038/s41598-021-98555-y (PMC8476564; doi:10.1038/s41598-021-98555-y)
Supplement: Supplementary file 1 — Supplementary Information. [file 41598_2021_98555_MOESM1_ESM.docx]

In vitro and in vivo comparative study of a novel ^68^Ga-labeled PSMA-targeted inhibitor and ^68^Ga -PSMA-11

Huanyu Chen ^1,2,3^, Ping Cai ^2,4^, Yue Feng ^1,2,3^, Zhanliang Sun ^1,2,3^, Yinwen Wang ^2,4^, Yue Chen ^1,2,3^, Wei Zhang^1,2,3^, Nan Liu ^1,2,3,^*, Zhijun Zhou^1,2,3,4,^*

^1^The Department of Nuclear Medicine, Affiliated Hospital of Southwest Medical University, Jiangyang District, Luzhou, Sichuan, China

^2^Nuclear Medicine and Molecular Imaging Key Laboratory of Sichuan Province, Jiangyang District, Luzhou, Sichuan, China

^3^Academician (Expert) Workstation of Sichuan Province, Jiangyang District, Luzhou, Sichuan, China

^4^Department of Pharmaceutics, School of Pharmacy, Southwest Medical University, Jiangyang District, Luzhou, Sichuan, China

Correspondence: zhouzjiang@gmail.com, everydayhappy815@163.com

**Synthesis**

1. The synthesis of **b-3**

To a solution of **b-1** (6.0 g, 20.3 mmol) and TEA (4.11 g, 40.7 mmol) in DCM (60 mL) was added Triphosgene (2.00 g, 6.71 mmol) at room temperature and stirred at 0°C. The mixture was stirred at room temperature for 1 h. **b-2** (5.30 g, 14.2 mmol) and TEA (1.44 g, 14.2 mmol) was added the above solution at room temperature and stirred at room temperature for 16 h. The mixture was poured into ice water, extracted with EtOAc, washed with brine and dried over Na_2_SO_4_. The organic layer was concentrated in *vacuo* and purified by column chromatography (petroleum ether/EtOAc = 5/ 1) to give **b-3** (7.5 g, 59.4% yield) as a yellow oil.

1. The synthesis of **b-4**

A mixture of **b-3** (4.0 g, 6.44 mmol) and Pd/C in EtOAc (100 mL) was stirred at 1atm of H_2_ for 16 h. The mixture was concentrated in *vacuo* and purified by column chromatography (EtOAc = 1/ 1) to give **b-4** (2.7 g, 86.1% yield) as a bark oil.

1. The synthesis of **b-6**

A mixture of **b-4** (2.5 g, 5.13 mmol), **b-5** (1.32 g, 4.50 mmol) and K_2_CO_3_ (1.42 g, 10.3 mmol) in DMF (40 mL) was stirred at 60°C for 16 h. The mixture was poured into ice water, extracted with EtOAc, washed with brine and dried over Na_2_SO_4_. The organic layer was concentrated in *vacuo* and purified by column chromatography (petroleum ether/EtOAc = 1/ 1) to give **b=6** (2. g, 77.7% yield) as a yellow solid.

1. The synthesis of **b-8**

To a solution of **b-7** (0.9 g, 3.63 mmol) in DMF (20 mL) was added HATU (1.65 g, 4.35 mmol) and stirred at room temperature for 0.5 h. **b-6** (2.70 g, 4.0 mmol) and DIPEA (0.94 g, 7.26 mmol) was added the above solution and stirred at room temperature for 16 h. The mixture was poured into ice water and extracted with DCM, washed with brine and dried over Na_2_SO_4_. The organic layer was concentrated in *vacuo* and purified by column chromatography (DCM /MeOH= 30/ 1) to give **b-8** (2.2 g, 73.2% yield) as a yellow oil.

1. The synthesis of **b**

A mixture of **b-8** (2.0 g, 2.15 mmol) and N_2_H_4_.H_2_O (1 mL) in EtOH (50 mL) was stirred at 90°C for 8 h. The mixture was concentrated in *vacuo* and purified by column chromatography (DCM /MeOH= 10/ 1) to give **b** (0.89 g, 51.7% yield) as a yellow solid.

1. The synthesis of **TM-1**

To a solution of **a** (0.5 g, 0.71 mmol) in DMF (20 mL) was added HATU (0.41 g, 1.07 mmol) and stirred at room temperature for 0.5 h. **b** (0.60 g, 0.75 mmol) and DIPEA (185 mg, 1.43 mmol) was added the above solution and stirred at room temperature for 16 h. The mixture was poured into ice water and extracted with DCM, washed with brine and dried over Na_2_SO_4_. The organic layer was concentrated in *vacuo* and purified by column chromatography (DCM /MeOH= 20/ 1) to give **TM-1** (0.50 g, 47.2% yield) as a yellow solid.

1. The synthesis of **SC691**

A mixture of **TM-1** (0.4 g, 0.27 mmol) and HCl (gas)/ 1,4-dioxane (20 mL) was stirred at room temperature for 16 h. The mixture was concentrated in *vacuo* and purified by Prep-HPLC to give **SC691** (115 mg, 39.1% yield) as a yellow solid. MS (ESI): [M+H]^+^=1093.2, ^1^H NMR (CD_3_OD, 400 MHz), δ 7.860 (d, *J* = 8.4 Hz, 2H), δ 7.184 (q, *J* = 3.6 Hz, 2H), δ 4.329 (m, 2H), δ 3.983 (t, 1H), δ 3.800 (m, 6H), δ 3.542 (m, 7H), δ 3.252 (m, 15H), δ 2.584 (m, 4H), δ 2.216 (m, 1H), δ 2.216 (m, 3H), δ 1.755 (t, 3H), δ 1.591 (m, 7H), δ 1.499 (m, 2H).


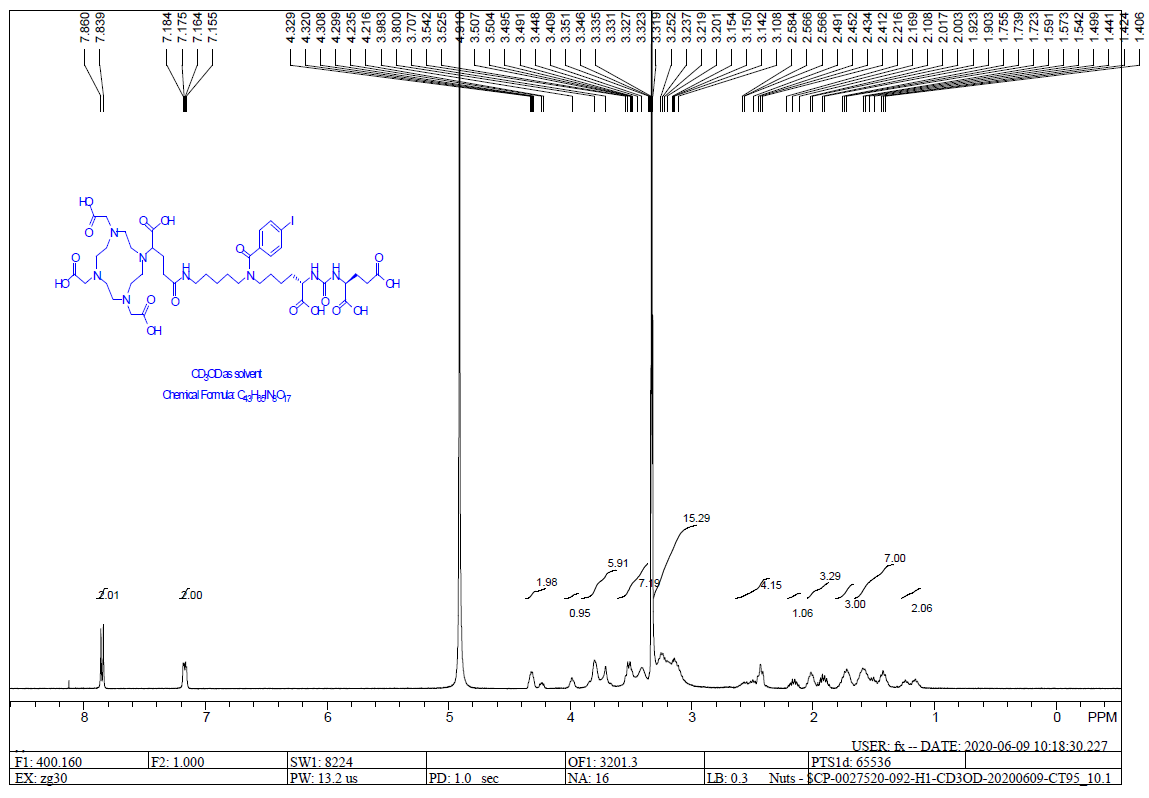


Figure S1. ^1^H NMR of SC691 (400 MHz).


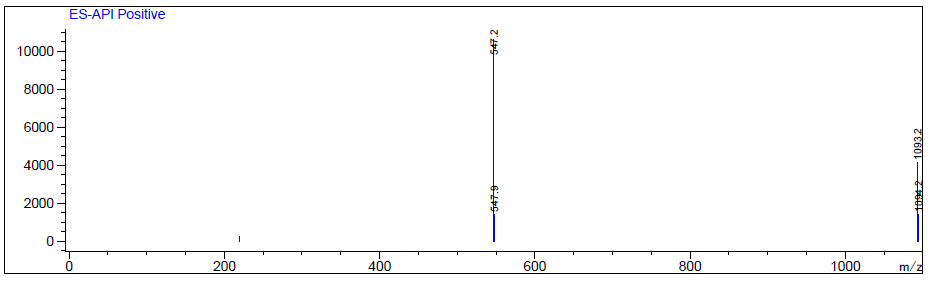


Figure S2. Mass Spectroscopy of SC691 (ESI) at retention time 1.33 min.

Micro-PET/CT Imaging


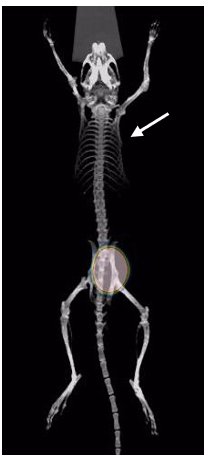


Figure S3. Blocking experiment for ^68^Ga-SC691 in LNCaP inoculated NOD/SCID xenografts. Whole-body coronal micro-PET/CT images of an NOD/SCID male mouse bearing LNCaP tumor xenografts (white arrow).


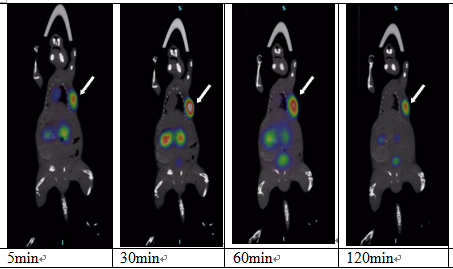


Figure S4. Whole-body coronal micro-PET/CT image of an NOD/SCSID male mice bearing LNCaP tumor xenografts (white arrow). The tumor targeting efficacy of ^68^Ga-SC691 was demonstrated by time-dependent static scan at 5 min, 30 min, 60 min, 120 min post injection of ^68^Ga-SC691. Approximately 1.9 MBq/ mouse was injected.
